# Evaluation of three methods of suture for skin closure in total knee arthroplasty: a randomized trial

**DOI:** 10.1186/s12891-021-04627-5

**Published:** 2021-08-30

**Authors:** Rodrigo Barreiros Vieira, Gustavo Waldolato, João Sequeira Fernandes, Thiago Gontijo de Carvalho, Pedro Augusto Maciel Moreira, Guilherme Barbosa Moreira, Jorge Suman Vieira

**Affiliations:** Department of Orthopaedics, Hospital Universitário Ciências Médicas-Faculdade de Ciências Médicas de Minas Gerais, Rua dos Aimorés, 2896- Santo Agostinho, MG 30140-073 Belo Horizonte, Brazil

**Keywords:** Total knee arthroplasty, Suture, Skin closure

## Abstract

**Background:**

There are several studies comparing techniques and different materials, yet the results are not unanimous. We compared three methods of skin closure in total knee arthroplasty (TKA), including suture with single stitches and unabsorbable *MonoNylon®*, as well as continuous subcuticular suture with *Monocryl®* or barbed *Stratafix®* absorbable suture.

**Methods:**

A prospective, randomized study was conducted with 63 patients undergoing TKA between March 2016 and December 2016. Patients were divided into three groups: traditional suture *MonoNylon®* (n 22), subcuticular continuous suture with *Monocryl®* (n 20), and another barbed with *Stratafix®* (n 21). The closure time, length of wire used, pain intensity, possible complications, and cosmeses were evaluated.

**Results:**

Subcuticular continuous suture using *Monocryl®* was superior to traditional suture using *MonoNylon®* as less thread was used (*p* 0.01) and a better cosmetic effect was achieved (*p* < 0.01), which was equal to *Stratafix®* aspects analyzed (*p* > 0.05). Complications were observed mostly in patients who used *Stratafix®*.

**Conclusions:**

This study concluded that the subcuticular suture with absorbable monofilament *Monocryl®* proved to be advantageous compared to the others because it presented results equal to the barbed *Stratafix®*, however with fewer complications. Furthermore, *Monocryl®* was shown to be equal or superior to traditional *MonoNylon®* suture regarding in relation pain intensity, aesthetic result, and effective cost.

**Trial registration:**

WHO ICTRP identifier RBR78dh5d. Retrospectively registered: 07/29/2020.

## Background

Total knee arthroplasty (TKA) is increasingly performed for the treatment of symptomatic gonarthrosis, with a projection of more than 600 % increase in the number of surgeries in less than three decades and an expectation of 3.5 million annual procedures by 2030 in the United States [1]. This rapid increase in the number of surgeries is due to the aging of the population and to the excellent results achieved by TKA, leading to rates close to 90 % of satisfaction according to patients [2]. To a large extent, the improvement in the positive results is related to a better understanding of the biomechanics of the knee, to the improvement of the components of the prosthesis, and also to the technique used in all stages of the surgical procedure, including the closure of the skin.

Various materials can be used for skin closure by means of different techniques, with the purpose of minimizing complications such as scar pain, dehiscence, and infections, besides reduction in surgical time and aesthetic improvement. Stitching with simple suture and use of unabsorbable suture is the most widespread method since it is the simplest and has a low cost. Other techniques such as subcuticular suture, with unabsorbable or absorbable suture, and closure with staples are described and widely used in practice [3]. In the literature, there are several studies comparing techniques and different materials [4–6], yet the results are not unanimous.

In recent years, the barbed wire closure presents as a new option, with the justification of less time spent and cosmetic improvement of the scar. These threads increase the ability to approach and maintain the edges in soft tissues, minimizing episodes of dehiscence, in addition to avoiding the need for nodes, consequently visually improving the scar. The barbed wire suture has been accepted in plastic surgery [7], gynecology [8], urology [9], and general surgery. In the field of orthopedics, Gililland et al., in 2012, presented slightly favorable results with the use of this material in the closure of the TKAs in comparison with suture with simple points [10]. Positive findings were also found by other researchers [11]. However, other studies have not demonstrated the superiority of this method in comparison to others, especially its safety, leading to doubts as to its use in TKAs [12–14].

The present study aimed to compare three methods of skin closure in TKA, namely suture with single suture using unabsorbable *MonoNylon®*, as well as continuous subcuticular suture with *Monocryl®* absorbable or *Stratafix®* barbed.

## Methods

This is a prospective, and randomized study including patients who underwent TKA at the University Hospital in Brazil between March 2016 and December 2016.

All patients with gonarthrosis treated at the knee surgery outpatient clinic of the University Hospital participated in the study. The inclusion criteria were as follows; patients with primary or secondary gonarthrosis with indication of TKA, aged 45 to 85 years, both sexes. Patients with previous history of knee surgery, patients with inflammatory joint diseases, smokers (at least 1 cigarette per day (2)), alcoholics (consumption of 15 doses / week for men and 10 doses / week for women (1)), with hypoalbuminemia (below 3.5 mg / dl (3)), and anemia (hemoglobin ≤ 10 mg / dl) were excluded. Of the 68 patients who met the inclusion criteria, five were excluded, leaving 63 patients in the study.

Participants were evaluated at the institution’s knee surgery outpatient clinic. In this analysis, demographic data (sex, age, laterality, body mass index [BMI]) were collected and randomized by draw of a sealed brown envelope containing three cards referring to each of the groups: group 1 (single suture), 22 patients underwent single-stitched interrupted sutures and use of monofilament monofilament *MonoNylon®* 3 − 0 (Ethicon®, Johnson & Johnson); group 2 (intradermal suture), 20 patients underwent continuous intradermal suture with monofilament absorbable monofilament *Monocryl®* Polytechnique 25) 3 − 0 (Ethicon®, Johnson & Johnson); and group 3 (barbed wire suture), 21 patients underwent continuous intradermal suture using *Stratafix®* unidirectional PGA-PCL barbed monofilament barbed wire (Ethicon®, Johnson & Johnson). The surgical procedures were scheduled up to 2 weeks after randomization by sealed envelope. One of the researchers (GW) was responsible for randomization and another researcher (GBM) enrolled participants.

The preparation of the surgical procedure, including antibiotic therapy (Cephalosporin 1º generation) 30 min before anesthetic induction and anesthesia (spinal anesthesia + sedation) was uniform for all patients, according to the routine of service. A pneumatic tourniquet was used in all patients, besides an anterior incision 17 cm long, with access to the joint cavity by medial parapatellar arthrotomy, eversion, and lateral dislocation of the patella.

After implantation of all components of the prosthesis (Modular III®, MDT, Brazil), closure of the arthrotomy with absorbable interrupted suture *Vicryl®* 0 (Ethicon®, Johnson & Johnson) and of the subcutaneous tissue with absorbable interrupted suture *Vicryl®* 2.0 (Ethicon®, Johnson & Johnson), the skin was sutured using the technique defined by prior randomization. All surgical procedures were performed by the same surgeon (RBV). The skin closure was performed with the knee in extension, in a unidirectional direction from proximal to distal. The beginning of the skin closure until its term was timed the time spent in making the suture and at the end, the length of thread used was gauged.

The incision was dressed with sterile gauze and Micropore surgical tape (3 M Company). The dressing were changed daily for 14 days and then removed.

The postoperative procedures of analgesia, anticoagulation, antibiotic therapy, hospital discharge, dressing method and wound care and guidelines were also the same for all.

Patients were reevaluated for this study after 2 and 12 weeks in terms of wound pain intensity according to the visual analogue scale (where 10 = maximum intensity pain and 0 = no pain) and changes in wound healing (infectious, allergic signs, and dehiscence). Additionally, in the 12th week, patients were evaluated for the cosmesis degree of the scar according to the Stone Brooks Scar scale for surgical wound cosmesis [15] (Table 1).

**Table 1:** Stony Brook Scar Evaluation Scale

Scar category		Points
Width	> 2 mm	0
	≤ 2 mm	1
Height	Elevated/depressed in relation to suronding skin	0
	Flat	1
Color	Daker than surrounding skin	0
	Same color or light than surrounding skin	1
Hatch marks/suture marks	Present	0
	Absent	1
Overall apparence	Poor	0
	Good	1

* Total score sum of individual scores; range, 0 (worst) to 5 (best)

The data were tabulated in Microsoft Excel® and the results were presented in tables and measurements. A descriptive and inferential analysis of the results was performed. To guarantee the accuracy of the comparisons, the homogeneity of the samples was verified using Shapiro-Wilk test. The comparisons were performed using the Tukey test. The variables were analyzed by the R software through the TukeyHSD function, where the model is the ANOVA. A significance level p < 0,05 was considered in all these comparisons.

## Results

In the demographic analysis of the studied sample, 73 % (*n* = 46) of the population was female and 27 % (*n* = 17) was male. The mean age of the patients was 68 years (range: 45–81 years). Regarding BMI, 46.1 % of the patients had a BMI > 30, considered obese, 42.9 % between 25 and 30, considered overweight, and only 11.1 % within the normality index. Demographic data between groups are summarized in Table 2.

**Table 2:** Compared demographic data between groups

Group	n	Sex F:M	Age (mean)	BMI<25 25–30 >30	
Simple	22	18:4	68.95		1 11 10
Monocryl®	20	13:7	69.75		3 7 10
Stratafix®	21	15:6	64.33		3 9 9

*n* number of patients *F* famele *M* male *BMI* Body mass index

### Closing time

Comparing the types of sutures in relation to the time of closure of the skin, it was observed that the unabsorbable simple suture group recorded a time significantly inferior to the others (*p* < 0.05), with a mean of 7.40 min compared to 8.90 and 9.00 min in the *Monocryl®* and barbed *Startafix®* groups, respectively (Table 3).

**Table 3:** Analysis of time spent, in minute, on skin closure

Group	Min.	Max.	Mean	±SD	95 %CI	Group x Group	*p*^*1*^
Simple®	7.03	16.00	7.40	1.88	[6.61–8.19]	Simple x Stratafix®	*0.01*
Monocryl®	7.00	12.30	8.90	1.44	[8.23–9.57]	Monocryl® x Simple	*0.01*
Stratafix®	5.00	11.35	9.00	1.77	[8.15–9.87]	Stratafix® x Monocryl®	*0.98*

*Min.* minimum; *Max.* maximum; *SD* Standard deviation; *CI* Confidence interval

^1^Tukey Test

### Wire length used

Regarding the length of the wire used for suturing the skin, the unabsorbable simple suture required a significantly longer wire length than that used for both the absorbable *Monocryl®* and the barbed *Stratafix®* sutures (*p* < 0.05). However, when the barbed and *Monocryl®* wire groups were compared, no significant difference was observed (Table 4).

**Table 4:** Comparison of the length of suture, in centimeter, used between groups

Group	Min.	Max.	Mean	±SD	95 %CI	Group x Group	*p*^*1*^
Simple®	38.30	118.00	83.53	17.95	[75.57–91.49]	Simple x Stratafix®	*0.00*
Monocryl®	19.00	48.00	28.25	8.67	[24.19–32.28]	Monocryl® x Simple	*0.00*
Stratafix®	22.00	33.00	27.33	2.69	[26.11–28.55]	Stratafix® x Monocryl®	*0.35*

*Min.* minimum; *Max.* maximum; *SD* Standard deviation; *CI* Confidence interval

^1^Tukey Test

### Intensity of pain

Regarding the intensity of pain in the first postoperative evaluation at 2 weeks, the barbed *Stratafix®* suture group presented more intense pain when compared to the unabsorbable simple suture group (*p* < 0.01). (Table 5)

**Table 5:** Analysis of pain intensity between groups at 2- and 12-weeks post-operative

Time^*^	Group	Min.	Max.	Mean	±SD	95 % CI	Group x Group	*p*^*1*^
	Simple	0	8	3.87	2.21	[2.88–4.84]	Simple x Stratafix®	*0.01*
2	Monocryl®	1	10	5.25	2.40	[4.12–6.37]	Monocryl® x Simple	*0.11*
	Stratafix®	3	9	6.00	1.92	[5.12–6.87]	Stratafix® x Monocryl®	*0.51*
12	Simple	0	6	2.00	1.74	[1.23–2.77]	Simple x Stratafix®	*0.09*
	Monocryl®	0	7	2.75	2.36	[1.64–3.85]	Monocryl® x Simple	*0.53*
	Stratafix®	0	9	3.48	2.58	[2.30–4.65]	Stratafix® x Monocryl®	*0.55*

*Min.* minimum *Max.* maximum *SD* Standard deviation *CI* Confidence interval

*Weeks ^1^Tukey Test

Regarding the second evaluation at 12 weeks, there was a reduction in pain sensation in all three groups; however, the barbed *Stratafix®* suture group reported more severe pain. There was no significant difference between the groups (*p* > 0.05).

### Analysis of surgical wound cosmesis

In the evaluation of the surgical wound cosmesis, the mean Stone Brook Scar score of the unabsorbable suture was 1.91 whereas that of the subcuticular *Monocryl®* and barbed totaled 3.30 and 3.00, respectively. There was a significant difference in the cosmesis score between groups, with Post-hoc tests revealed that a significant difference existed between the unabsorbable suture and *Monocryl®* groups as well as between the unabsorbable *MonoNylon®* suture and barbed *Stratafix®* suture groups (Table 6).

**Table 6:** Analysis of the cosmesis score between groups, according Stony Brook Scar scale

Group	Min.	Max.	Mean	±SD	95 %CI	Group x Group	*p*^*1*^
Simple®	0	4	1.91	1.23	[1.36–2.45]	Simple x Stratafix®	*0.00*
Monocryl®	1	5	3.30	1.03	[2.82–3.78]	Monocryl® x Simple	*0.01*
Stratafix®	0	5	3.00	1.30	[2.41–3.60]	Stratafix® x Monocryl®	*0.70*

*Min.* minimum; *Max.* maximum; *SD* Standard deviation; *CI* Confidence interval

^1^ Tukey Test

### Complications

Regardless of the type of suture performed, no patient presented with an allergic reaction to the thread used. In addition, only one patient, who was in the barbed *Stratafix®* suture group, presented with a superficial infection. Ten patients had a slight dehiscence, less than 20 % of the operative wound length, of which four belonged to the subcuticular *Monocryl®* suture group and six to the barbed *Stratafix®* suture group. Patients submitted to single closure unabsorbable *MonoNylon®* did not present with any postoperative complications (Table 7).

**Table 7:** Analysis of the complication between groups

Group	n	Allergic reaction	Superficial infection	Slight dehiscence
Simple	22	-	-	-
Monocryl®	20	-	-	4
Stratafix®	21	-	1	6

*n* number of patients.

## Discussion

All aspects related to TKA are constantly evolving, with the purpose of improving patient satisfaction and reducing complications. In the last decade, many studies have attempted to compare the best mode of cutaneous closure in arthroplasties; however, the heterogeneity of the methods used and the lack of uniformity of the results led to the realization of this prospective and randomized study. The present study showed that there was no difference in the parameters searched between subcuticular sutures, whether with absorbable *Monocryl®* or barbed *Stratafix®*; however, they were superior to the suture with simple points, especially regarding the cosmetic aspect.

The present study included mostly female patients, corroborating with other studies on knee arthroplasty [11, 16], which was expected due to the higher prevalence of gonarthrosis in women [17]. Also, about half of the patients were obese, who are more likely to have complications in the healing of operative wounds [18]. However, these patients were homogeneously distributed among the evaluated groups, not interfering with the results. The intradermal suture with barbed wire group was composed of patients younger than the other groups (p 0.01); however, the authors did not believe that the small difference (mean: 5 years younger) influenced the results of the parameters analyzed.

The longer the time spent in surgery increases the morbidity of the procedure; therefore, it is always important to use techniques that minimize surgical time. Following this prerogative, a shorter time spent closing the skin can be of great help. Gililland et al. Reported a significantly shorter closure time with subcuticular suture using *Stratafix®* compared to single suture [10], as did other authors [16, 19, 20]. Smith et al. found that the use of subcuticular suture starting at the center of the wound and directing to the distal and proximal region of the wound made simultaneously by two professionals is a justification for the shorter closure time with subcuticular sutures with *Stratafix®* versus traditional unabsorbable sutures. However, Maheshwari et al. showed that there was no difference in time spent using subcuticular sutures with *Stratafix®* or traditional single stitches in knee arthroplasties [21]. We found that the closure time using simple suture was faster than the subcuticular ones with absorbable *Monocryl®* or barbed *Stratatifx®*. However, despite this being statistically significant, on average the time was only about 90 s shorter, a difference that we considered not to influence the total time of arthroplasty.

The amount of wire used between the three different sutures was found to differ between groups. The continuous subcuticular techniques with either with *Monocryl®* or with *Stratafix®* used on mean a little more than 30 % of the length used in single suturing. However, the specific value of wire used is appreciably lower with the use of *MonoNylon®* and especially *Monocryl®* compared to barbed *Stratafix®*, based on the commercial values ​​of each suture in the Brazilian market. The cost of the converted *MonoNylon®* is USD $0.04 / cm of wire, *Monocryl®* is USD $0.09 / cm, while the *Stratafix®* is USD $0.83 / cm. Taking into account the average wire used in the study for each technique, $2.54 was spent on the skin suture of each patient with *Monocryl®*, $3.34 per patient with *MonoNylon®* thread, and $22.68 per patient with sutures using *Stratafix®*. Ting et al. also showed a significantly higher cost in cutaneous closure with barbed wire compared to control (53 vs. 9 USD, p < 0.01) [22]. Elmallah et al. clearly showed the highest specific cost occurred when using barbed wire [23].

Studies have shown that the use of barbed wire in skin sutures leads to a greater risk of adverse effects compared to other materials. However, few studies have specifically evaluated the superficial closure, as proposed in this study. According to a review of the literature presented by Fauor et al., only four studies compared the complications in skin closure with barbed wire and conventional techniques; these authors observed that most of the studies used the barbed wire in the deep closures [20]. Campbell et al. presented higher rates of superficial and deep infection (p˂0.01) in patients undergoing skin stricture with barbed wire compared to staples, and two of the patients in the barbed wire group required complete revision of the arthroplasty [24]. Chawla et al. also concluded that patients submitted to barbed wire closure have a higher risk of superficial infection [25]. Corroborating with these findings, in our study, the patients in the barbed suture group had pain of greater intensity in the postoperative period, besides one patient who had superficial infection, and most of the dehiscences affected the patients of this group. The overtightening of barbed suture may predispose to less mobility between the cutaneous edges of the operative wound, resulting in greater tension during flexion and extension of the knee, leading to greater pain intensity compared to the other methods studied. It may also be a possible cause of ischemic cutaneous necrosis, resulting in dehiscence and superficial infection according to Campbell et al.[24].

The cosmetic appearance of the scar after any surgery is an important aspect of patient satisfaction, especially in women. Cosmetic evaluation, although very subjective, generally considers the color, width, and thickness of the scar, which, by the third month, shows a strongly predictive aspect of the long-term appearance, as concluded by Quinn et al. [26]. We used these concepts to guide the cosmesis analysis of the patients in the present study, where we evaluated the third month after surgery through the Stony Brook Scar scale that scores with the presence or absence of the following parameters: width greater than 2 mm, elevation or depression, discoloration, suture or staple marks, and overall poor appearance, with 5 being the maximum cosmetic value and 0 being the worst evaluation [15]. Gililland et al. [10] and Ting et al. [22] did not find a difference between the traditional and barbed wire techniques using a tool to evaluate the cosmetic appearance of the surgical scar to the level of patient satisfaction using the Hollander Wound Evaluation Score, which uses parameters close to that of Stony Brook Scar scale. In the present study, we found better cosmetic results in the subcuticular suture groups, either with *Monocryl®* or barbed *Stratafix®* wire, compared to the single interrupted suture with *MonoNylon®* group. The presence of darker staining and marks are very common aspects of the simple unabsorbable interrupted sutures that possibly led to a worse evaluation of the cosmesis in relation to the other two techniques.

This study has limitations that should be considered. First, the casuistry was small, so the conclusions reached can generate some insecurity. Secondly, the experience of the surgeon who performed the cutaneous closure in the conventional technique with simple suture was greater than in the other techniques, which may have led to bias in the evaluation of the closing time. In addition, use of unidirectional wires, which is different to several other authors who used two-way barbed wire, and the use of two surgeons during skin closure, as reported by Smith et al. [12] could be a possible limitation. And finally, the cosmetic evaluation had very subjective aspects, which could result in an inaccurate analysis of the results.

## Conclusions

This study concluded that continuous subcuticular suture with absorbable monofilament *Monocryl®* proved advantageous in relation to the barbed *Stratafix®* and monofilamentar unabsorbable *MonoNylon®* single suture, which presented with equal or superior results in pain intensity, esthetic result, and cost effectiveness.

## Data Availability

The datasets used and/or analysed during the current study are available from the corresponding author on reasonable request.

## Abbreviations

TKA: Total Knee Arthroplasty
BMI: Body Mass Index
